# Food security under water scarcity: a comparative analysis of Egypt and Jordan

**DOI:** 10.1007/s12571-022-01310-y

**Published:** 2022-09-16

**Authors:** Maria Christoforidou, Gerlo Borghuis, Chris Seijger, Gerardo E. van Halsema, Petra Hellegers

**Affiliations:** Water Resources Management Group, Wageningen University and Research, Wageningen, The Netherlands

**Keywords:** Water scarcity, Food security strategies, Egypt, Jordan, 2008 world food crisis

## Abstract

Although there seems enough water available for our global food needs, there are large areas with growing water scarcity. Food security in these water scarce areas cannot be met through self-sufficiency. The only option is to become more dependent on food imports which is increasingly risky due to volatility in production and food prices. Before 2008, declining food prices and increasing global cereal production favoured the food import strategy. The 2008 world food crisis represented a shock to this strategy and renewed attention was paid to the self-sufficiency strategy. The aim of this paper is to compare the food security strategies of Egypt and Jordan, two water-stressed, increasingly populated, oil-poor countries, pre and post 2008, by means of a food-water analytical framework using FAOSTAT data. Findings show that Egypt and Jordan have many similarities in their food security situation as both are highly dependent on food imports (Egypt 50%, Jordan 95%), and both have a reduced capacity to absorb future price increases. As food imports are inevitable under the water scarce context of Egypt and Jordan, it is important to focus on how to cope with volatilities. Our analysis shows that Jordan has better absorbed the costs of rising food imports than Egypt and that Egypt is trapped by its high domestic cereal production. Having revealed the limited options available to water-scarce countries for food security, we discuss the potential of grain reserves to cope with future price hikes and production shocks.

**Supplementary Information:**

The online version contains supplementary material available at 10.1007/s12571-022-01310-y.

## Introduction

Food security is one of the great challenges of our time and is a central concern of the global agenda towards 2030 (Mechiche-Alami et al., 2021; UN, 2016). Worldwide, processes of population growth, climate change, changing diets and urbanization are exerting rising pressures on available water resources. Given the economic and environmental limitations on increasing water supplies, water scarcity is becoming an ever more pressing reality (Molden, 2007). Since irrigated agriculture is the world’s largest water user, water scarcity particularly impacts agricultural production, and thus food security (FAO, 2020).

Food security at the national level can be obtained through two main strategies (or a mixture of the two): (i) domestic food production for self-sufficiency, (ii) food purchases on global markets (imports) (Clapp, 2017; Smith & Glauber, 2020). Before the 2008 world food price crisis, global food prices steadily declining (Molden, 2007), favouring the adoption of the trade strategy. The declining food prices were a big pull (and push, from the IMF and WTO perspective) factor for the adoption of the food import strategy. Central to the global trade strategy is the notion that well-organized markets and trade will enable all to obtain sufficient food and attain food security (Betge, 2016).

Since the 2008 world food crisis, price volatility has starkly increased (Tadasse et al., 2016), exposing the risks of the global trade strategy. Price volatility, if large and unexpected, might have significant impacts for food security. Especially for low- and middle-income countries that largely dependent on staple food imports, increased prices put a large claim on government budgets, and they thus lead to deteriorating public finances and inflation that has profound effect on poor communities (FAO et al., 2011; Nin-Pratt et al., 2017). In turn, governments are likely to try to absorb such price increases through subsidies and other fiscal measures that will lead to a further deterioration of the countries’ public finances (FAO et al., 2011).

After the 2008 food crisis, renewed attention was paid to the self-sufficiency strategy to counteract the vulnerability of trade strategy to price volatility (Clapp, 2017). The self-sufficiency strategy is reliant on domestic food production and thus countries have more control over food production and prices (Clapp, 2015; Moon, 2011). Among others, Russia, France, Egypt and China officially aimed at increasing their self-sufficiency, triggering a reorientation of national food security strategies (Clapp, 2017). This strategy is generally predominant around the globe. Indicative of the self-sufficiency predominance is that world trade in coarse grains amounted to a mere 11% and 14% of global production in 2006 and 2019, respectively (FAO, 2008, 2019).

Despite the political will for adopting the self-sufficiency strategy, countries sometimes are restricted by climate change (causing domestic production variability), delimited expansion room for agriculture and continued rising populations. As such, countries might orient to the world trade strategy, being exposed to vulnerability due to price hikes and volatility, and geopolitical turmoil. Price hikes and volatility has steadily increased due to: i) increasing urbanisation and rising segment of urban poor that need to be supplied with food; ii) rising population, and iii) rising food spending ratio of disposable income, which means that small price hikes in food have large effect on inflation and poverty. These factors also increasingly affect food exporting countries, leading them to quicker resort to restrict food exports in times of price hikes. During the 2008 crisis, global food prices spiked and main cereal exporters reacted with export bans, causing food prices to rise even further (Cardwell & Kerr, 2014). Geopolitical turmoil could also lead to trade disruptions both for importing and exporting countries (Clapp, 2017). Clapp (2015, 2017) therefore advocates for viewing the two strategies as a continuum.

The Middle East and North Africa (MENA) region is the largest net cereal importer and most water scarce region in the world (Lee et al., 2019). Food demands continue to increase due to high population growth rates while national water resources are no longer adequate to produce sufficient food to meet domestic demand (Lee et al., 2019; Nin-Pratt et al., 2017), effectively limiting the possibilities for the self-sufficiency strategy (Woertz, 2017) and increasing dependence on the global trade strategy. This dependence, in turn, exposes the region to price volatilities (Battat & Lampietti, 2011) and possible trade disruptions (Mrdalj & El Bilali, 2021). Thus, MENA countries are faced with tough choices and trade-offs for meeting food security and minimize their vulnerability.

Several studies assessed the interlinkages of water and food security in the MENA region (e.g. Antonelli & Tamea, 2015; Fathelrahman & Muhammad, 2016; Hameed et al., 2019; Larson, 2013; Terwisscha van Scheltinga et al., 2021). Other studies have focused on modelling past trends and projecting towards the future, discussing the interplays between food security and, among others, natural resource management (Abdelkader & Elshorbagy, 2021; Rosegrant & IMPACT Development Team, 2012; Sulser et al., 2011).Yet, these studies did not analyse to what extent food security strategies shifted to reduce the reliance on global food markets for food security in MENA countries. To do so, we focus on two MENA countries, Egypt and Jordan, two water-stressed, increasingly populated, oil-poor countries.

Water scarcity is a contextual reality and a starting point of this analysis, in which the food security question needs to be resolved. Egypt and Jordan are severely limited in their food security options as augmenting water resources to expand domestic agricultural production is not feasible. Both countries are located in closed river basins (FAO & Ihe Delft, 2020; Venot et al., 2008). Any rearrangement of agriculture will necessarily involve a re-ordering and reallocation of water between agricultural production sectors (particularly, cereals and high-value crops), as well as between agriculture and other economic sectors.

In this paper we analyse how the application of the global trade and self-sufficiency food security strategies under water scarcity have evolved in Egypt and Jordan before, during and after the 2008 world food price crisis. This pre–post analysis constitutes a valuable addition to earlier studies on global food prices and impacts on the Arab Spring (Soffiantini, 2020) and agricultural development in the wake of the Arab Spring (Woertz, 2017). Section 2 presents the analytical framework for this study. Section 3 presents the results of applying the analytical framework on the food security status of Egypt and Jordan as well as a comparative analysis between the two countries. The comparison reveals that despite many similarities (as food security in both countries depends to food imports and on the wider economy’s capacity to finance the required food imports), the outlook for the future is different (as countries’ economic capacity to cope with price volatilities and hikes is different). The paper ends with a discussion on the limitations of our analysis and the Covid-19 crisis (Sect. 4) and the key conclusions and recommendations for policy (Sect. 5).

## Materials and methods

### Food-water analytical framework

This study applied a food-water analytical framework to investigate changes in national food security strategies pre and post 2008 in countries with high degrees of water scarcity. Food security was assessed through food availability, food access and supply stability (ESA, 2006; Jones et al., 2013). The water resources base was assessed through water availability and the scope to enhance the water resources base by augmenting supply or by water savings (Siderius et al., 2021). The food-water analytical framework consists of quantitative and qualitative indicators that are explained in further detail in this section (Table 1).

**Table 1:** Food-water analytical framework

**Food security**	**Indicators**	**References**
Food availability (related to availability of food from both domestic production and imports)	Domestic food production quantities (Mtonnes), see Figs. 1 and 5 Domestic consumption per 1,000 people (tonnes/1,000 persons), see Figs. ii and vi in SI	Nicholson et al. (2021)
Food access (related to adequate resources for acquiring food, thus prices are indicative)	Food consumer price index (CPI) (%), see Figs. 2 and 6	Jones et al. (2013)
Stability (vulnerability) (related to the risk of losing food availability and access)	Self-sufficiency ratio (SSR) (%), Tables i, ii, iii and iv in SI Agricultural trade balance (million US$), see Figs. 3 and 7	Clapp (2017), FAO et al. (2013), Mechiche-Alami et al. (2021),
	Value of food imports over total merchandise exports (%), see Figs. 4 and 8	FAO et al. (2013), Mechiche-Alami et al. (2021).
**Water resources**		
Water availability	Rainfall (MCM) Surface water (MCM) Groundwater (MCM)	Qadir et al. (2007)
Water supply enhancement	Share of water resources that can be additionally exploited in a sustainable manner. Basin closure is when river flows fall short of commitments to sustain ecosystems	FAO (2012)
	Dependence ratio (%) Uptake of desalination	FAO (2022b)
Water savings^a^	Wet savings refers to a ‘real’ reduction in water losses as water not beneficially used elsewhere in a watershed Dry savings refers to a ‘paper’ saving of water losses as water losses were beneficially used elsewhere in a watershed	Cook et al. (2006), FAO (2012), Seckler (1996), Van Halsema and Vincent (2012)

^a^Wastewater reuse by agriculture might increase the agricultural water supply but cannot be considered a water supply enhancement as it does not increase water resources at the watershed level. Water savings and efficiency gains might increase the consumptive use of water at the expense of groundwater recharge and environmental flows, as these flows are considered losses at the farm level. As such, attention should be paid to whether water savings come from real losses, such as evaporation reduction, and not from losses that are beneficially used in the watershed (Cook et al., 2006; FAO, 2012; Seckler, 1996; Van Halsema & Vincent, 2012). Desalination can increase the overall water supply.

Food availability is defined as the availability of sufficient quantities of food of appropriate quality, including imports and food aid (ESA, 2006), indicated by the domestic food supply, including imports (Nicholson et al., 2021) of staples, fruits and vegetables. Domestic food supply was normalised for population (per 1,000 people), to account for the effect of population growth. Food access is defined as the physical and economic ability to secure food, and assessed using the food consumer price index or CPI as a proxy for food price volatility (Jones et al., 2013). The CPI is defined as ‘the price change between the current and reference periods of an average basket of goods and services purchased by households’ (FAO, 2022a). Stability of food supply is defined as the risk of disruptions in food availability and access (ESA, 2006), represented by the self-sufficiency ratio for cereals (defined by FAO et al. (2013) as the cereal import dependence ratio), the value of food imports to total merchandise exports (FAO et al., 2013; Mechiche-Alami et al., 2021), and the total agricultural trade balance. This last indicator represents the ability of the agricultural sector to finance food imports for food security.

Water availability was assessed using the Falkenmark indicator and the available renewable water resources per capita, thus expressing pressure exerted on water resources by the population (Qadir et al., 2007). The scope to enhance the water resources base was represented by a country’s current water use and whether there are additional water resources to be exploited (FAO, 2012), its dependence on water resources originating from beyond its national borders (i.e., the water dependence ratio from FAO, 2022b) and the scope for water savings and geopolitical considerations affecting access to surface water, sea water (for desalination) and/or groundwater (Cook et al., 2006; FAO, 2012; Seckler, 1996; Van Halsema & Vincent, 2012).

### Comparative case study: food security strategies in Egypt and Jordan

We selected the case study as our main research method due to its strength in analysing complex, multidimensional phenomena in context (Flyvbjerg, 2006). Our object of analysis was governmental food security strategies within their historical, political, agricultural and hydrological context. More specifically, we conducted a comparative case study (Steinberg & VanDeveer, 2012; White, 1987) of Egypt and Jordan to contrast different food security strategies in response to the world food price crisis in comparable contexts of water scarcity. It also permitted us to analyse the effects of different strategies in attaining food security in the face of disruptions in food imports and exports.

With careful case selection, findings from case study analysis can be generalized to a broader range of cases (Flyvbjerg, 2006; Gerring, 2016). Both Egypt and Jordan are examples of countries in the arid, water-stressed MENA region. The food security of both is largely dependent on food imports, and both countries were impacted by the world food price crisis and the Arab Spring. Their water resources for food production are extremely constrained, and neither country can rely on oil exports to finance food imports, unlike, for instance, Saudi Arabia or Iran (Yazdanpanah, 1994).

Our case analysis is structured by the food-water analytical framework and covers the 2000–2018 period. The findings rely on three data sources: time series data from AQUASTAT and FAOSTAT databases on food production, consumption and trade (FAO, 2022b, c); policy documents related to agriculture and water; and scientific reports and grey literature providing country-specific information on agriculture, water and food security. The crude quantitative data obtained from FAOSTAT are presented in the supplementary information (Table i-iii for Egypt and Table iv-vi for Jordan in S.I.). The policy documents, scientific report and grey literature documents were studied and policy reviews were conducted. The policy reviews were published as WaterPIP project reports^1^.

## Results

### Egypt case study

**Fig. 1:**
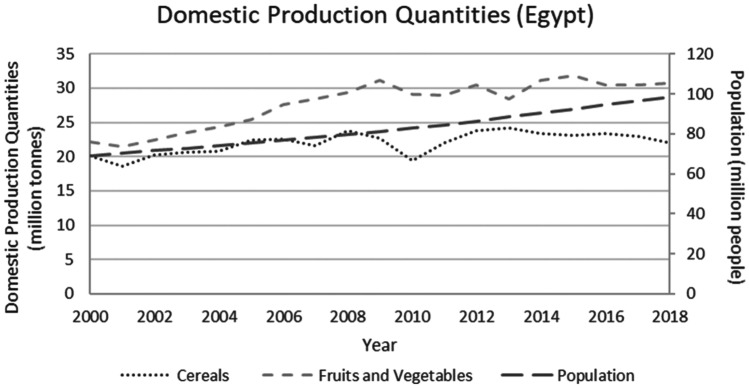
Domestic production of fruits and vegetables and cereals and population growth in Egypt, 2000-2018. Steadily growing population and increasing cereal and fruit and vegetables production pre 2008 and variations post 2008 (FAO, 2022c)

Egypt’s population grew by 40% between 2000 and 2018, from 68 to 98 million (Fig. 1). Total production of fruits and vegetables and of cereals increased as well. Between 2000 and 2008, Egypt managed to increase domestic cereal production at the same rate as population growth (Fig. 1). From 2008 to 2010, domestic cereal production declined by 18% and failed to follow the population growth trend. This was due to a combination of harsh winters and warm summer temperatures that declined production, and falling wheat prices that prompted Egyptian farmers to shift to other crops (WFP et al., 2013). As a result, the remaining cereal demand had to be met entirely by increased imports (see Fig. i in SI).

**Fig. 2:**
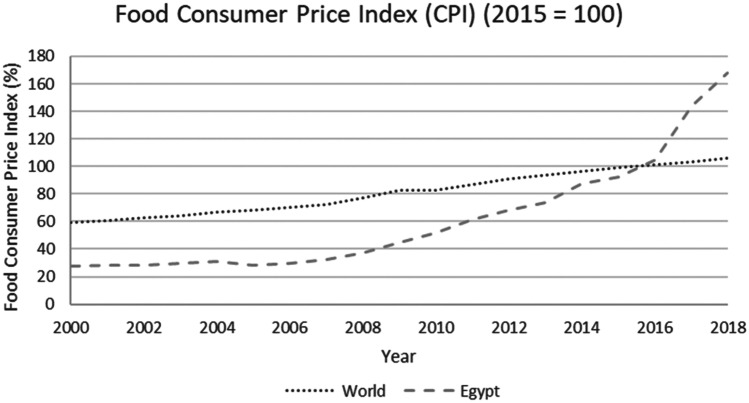
Food consumer price index in Egypt and the world, 2000–2018. Increasing food prices due to 2008 world food crisis and the change in monetary policy in 2016. (Source: FAO, 2022c)

The increases in imports required to meet rising food demand during the 2008 world food crisis were costly, resulting in steep domestic price hikes (Fig. 2). As a response to the world food crisis, Egyptian policymakers attempted to increase agricultural production, despite the limited opportunities for expanding and increasing domestic production due to water scarcity. In 2009, the country drafted a new agricultural strategy (MALR, 2009) defining self-sufficiency targets for specific crops in the Nile Delta while also promoting exports (mainly from production in newly reclaimed desert areas). Policies focused on increasing irrigation efficiency, expanding agricultural area and increasing land and water productivity (MALR, 2009).

In the short-term, and to ensure access to food and limit the impact of market volatility on food prices, Egypt implemented two main policies: (i) consumer food subsidies (Soffiantini, 2020; WFP et al., 2013) and (ii) production subsidies (McGill et al., 2015). Initially this strategy had the desired effect of restoring domestic production to pre-2008 levels by 2012/13 (see Fig. 1), and flattening the price index hike into a gradual rise between 2008 and 2016 (see Fig. 2). Domestic cereal consumption per capita remained relatively stable between 2008 and 2010, ranging from 449 tonnes/1,000 persons to 430 tonnes/1,000 persons respectively and was later increased until 2012 (478 tonnes/1,000 persons, see Fig. ii in SI). However, the strategy proved financially unsustainable, as it drained the national budget to purchase the food imports on the global market (Soffiantini, 2020, explained in next paragraph). This, in combination with pre-existing social and political unrest, triggered the Arab Spring in 2011 (Lybbert & Morgan, 2013; Soffiantini, 2020). During 2011 and with declining public reserves, Egypt was unable to import wheat and thus looked into the possibilities to increase state revenues through foreign investments and loans (Joya, 2017). In the years following the Arab spring, the policy discussions centred around budget cuts that included reductions in food and energy subsidies (Joya, 2017). Food subsidies were reduced from 1.8% of GDP in 2011 to 1.4% in 2016 (IMF, 2016). These, and the change from a fixed to a floating exchange rate (Samy-kamal, 2021), increased prices and initiated a decrease in domestic cereal consumption (see Fig. ii in SI) and production (see Fig. 1) from 2012 onwards. The food price index rose steeply after 2016, substantially exceeding the global average (see Fig. 2) and food accessibility declined.

**Fig. 3:**
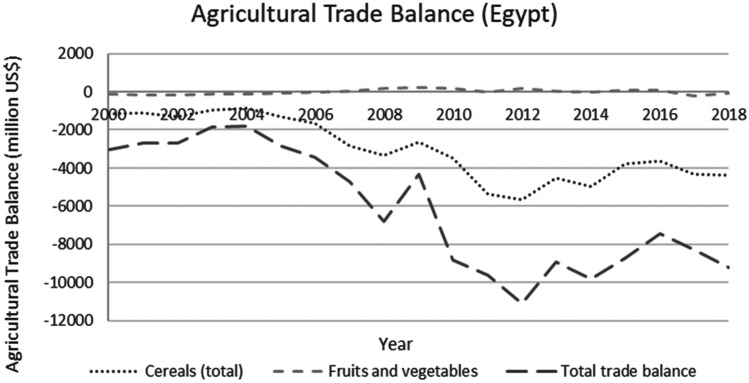
Agricultural trade balance of cereals, fruits and vegetables, and total in Egypt, 2000–2018. Decreasing total agricultural trade balance indicating the country’s inability to finance cereal imports from agricultural exports. Total agricultural trade balance includes the net value of imports and exports from the total agricultural products (FAO, 2022c)

**Fig. 4:**
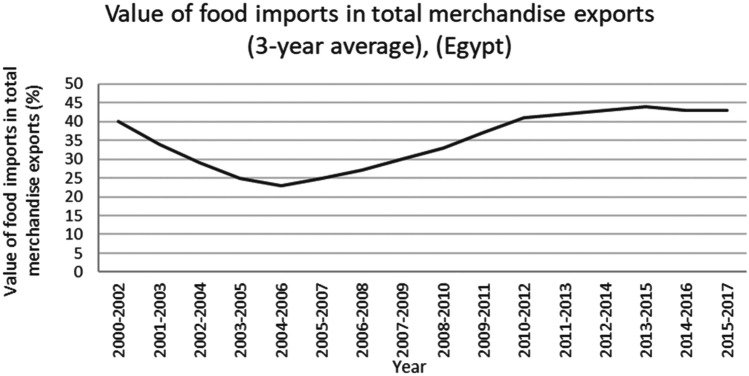
Value of food imports in total merchandise exports in Egypt, 2000–2017. A lower value for food imports (i.e., pre 2008) is more easily financed by the wider economy – in this case total merchandise exports – than when values of food imports are high (i.e., 2012 onwards) (FAO, 2022c)

The increased imports have had a major impact on Egypt’s agricultural trade balance (Fig. 3). Despite a more than 500% rise in exports of fruits and vegetables (see Fig. iii in SI), the agricultural trade balance remained insufficient to finance the rising cereal imports (Fig. 3). Egypt has thus to rely increasingly on other economic sectors to finance its import strategy. As shown in Fig. 4, the food imports value increased from 23% to around 45% of total merchandise exports. This means that the food imports represent nearly 50% of total export value of merchandise exports. As this ratio of food import value to total export value continues to rise, the more limited the buffer capacity of Egypt’s economy will be to accommodate further price increases.

Egypt has little opportunities to expand its water resources to grow food for domestic consumption. The country is very water scarce with a water availability of 596 m^3^/capita/year in 2017 (FAO, 2022b). The Nile provides 98% of Egypt’s renewable water resources (FAO, 2022c). Increases in Nile water inflows are highly unlikely, considering the irrigation developments in Sudan and Ethiopia and construction of the Grand Ethiopian Renaissance Dam (El-Nashar & Elyamany, 2018). Considering that the overall system efficiency was 80% (MWRI, 2017) and that the Nile Basin is a closed basin (FAO & Ihe Delft, 2020), the potential for efficiency improvements is limited. Groundwater extraction is increasing in the Nile Delta (Mohamed, 2020) and in some desert areas (Mohamed Ibrahem, 2019), which has raised questions regarding groundwater sustainability. Other than Egypt’s desalination efforts (Molle, 2019), possibilities for sustainable water supply enhancements and agricultural expansion for food production are severely limited (more information in Table vii in SI). Overall, there is thus very little additional water in Egypt to shift food security strategies (we return to this point in Sect. 3.3).

### Jordan case study

**Fig. 5:**
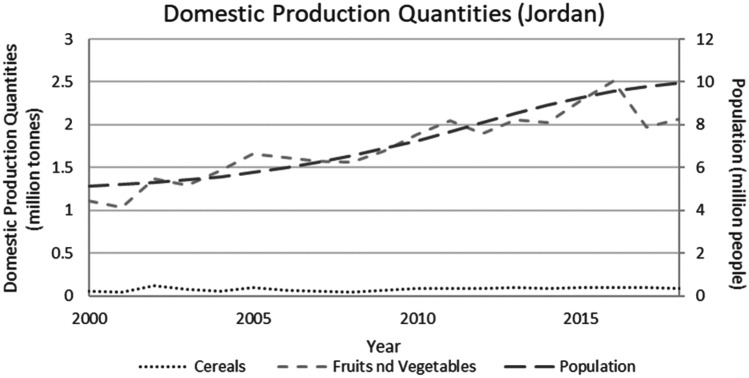
Domestic production of fruits and vegetables and cereals and population growth in Jordan, 2000–2018. Steadily growing population, increasing but limited domestic cereal production and steady increase of fruits and vegetables production (FAO, 2022c)

Jordan’s population almost doubled from 2000 to 2018, increasing from 5 million to some 10 million, due to demographic developments and a large influx of Syrian refugees after the onset of the Syrian civil war in 2011. Cereal production in Jordan is very limited (providing for less than 5% of total domestic consumption) (Fig. 5) although production did increase between 2000 and 2018 (see Fig. iv in SI). Thus, the country has met the population-growth-driven demand entirely by increased imports, making the country highly vulnerable to external shocks on the global food market.

In 2008, cereal imports decreased by 30% (see Fig. v in SI) due to rising prices and limited cereal availability in world markets. Due to the country’s high dependence on imports, the 2008 price spike directly affected Jordan’s food price index (around 20% increase for 2008–2014, Fig. 6). The doubling of the population, reduction of imports and price rises initiated a steady deterioration of food accessibility. Domestic cereal consumption per capita dropped from 414 tonnes/1,000 persons in 2007 to 243 tonnes/1,000 persons in 2010 (see Fig. vi in SI). Post-2010 consumption data indicate increased variability (see Fig. v in SI), likely associated with volatile global cereal prices.

**Fig. 6:**
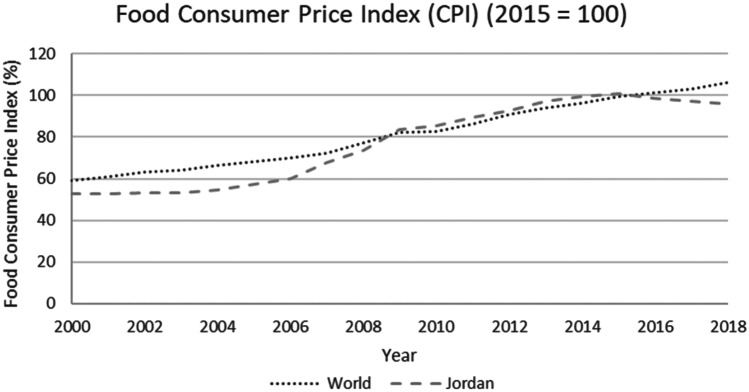
Food consumer price index in Jordan and the world, 2000–2018. Increasing food prices due to 2008 world food crisis, following a similar pattern as the global average, indicating that Jordan was able to avoid severe increases in food prices (FAO, 2022c)

Regarding fruits and vegetables, production followed a similar growth rate as population between 2000 and 2016 (Fig. 5), reflecting Jordan’s strategy to attempt to finance rising cereal imports by increasing high-value agricultural exports. The stated aim of the country’s 2020 agricultural policy was to increase economic output from agriculture, without increasing water use, by shifting to high-value, water-efficient crops (MOA, 2020). Exports of fruits and vegetables doubled between 2000 and 2013 yet declined after 2013 due to the Syrian civil war (see Fig. vii in SI), and domestic production declined from 2016 onwards (see Fig. 5). Together, these figures and numbers indicate a two-fold effect of the Syrian war on food security in Jordan: (i) increasing import requirements (due to the influx of refugees and population growth) and (ii) reducing export possibilities (due to the Syrian war and the closing of export routes to the European Union, Turkey and Russia).

**Fig. 7:**
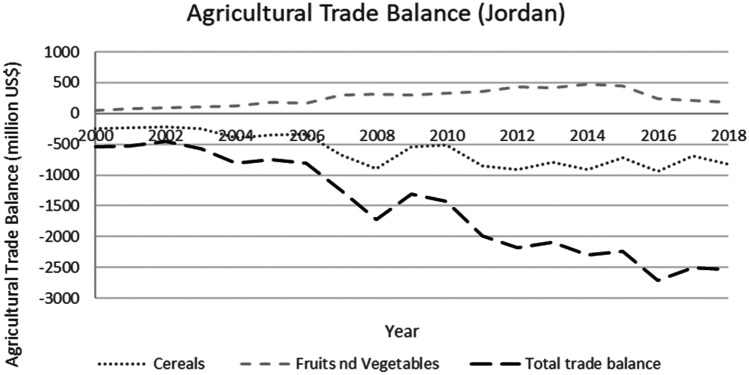
Agricultural trade balance of cereals, fruits and vegetables, and total in Jordan, 2000–2018. Decreasing total agricultural trade balance indicates the country’s inability to finance cereal imports from agricultural exports, with direct decreases during the world food price crisis (2008), Syrian war (2011) and closing of Jordan borders in 2016 (FAO, 2022c). Total agricultural trade balance used the same way as Egypt

**Fig. 8:**
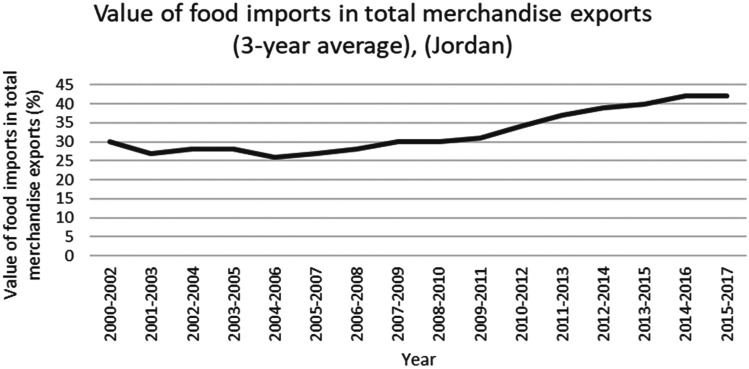
Value of food imports in total merchandise exports in Jordan, 2000–2017. Varying pre 2008 ability of Jordan to finance its food imports through total merchandise exports and decreasing ability post 2008 (FAO, 2022c)

Despite increased exports of fruits and vegetables between 2000 and 2013, the total agricultural trade balance remained negative (Fig. 7), indicating an inability to fund food imports with high-value agricultural exports. Jordan’s diminished ability to finance food imports was evident during the 2008 food price crisis and the second wave of price hikes in 2011 as the Syrian crisis started. The decrease observed in 2016 in the agricultural trade balance corresponds with the official closing of Jordan’s borders with Syria (Fig. 7). Under these conditions (2008 world food crisis, 2011 Syrian war and 2016 closing of Jordan’s borders), exports of fruits and vegetables were insufficient to offset the negative effects of the increased imports. Therefore, Jordan had to rely on earnings from other economic sectors to finance its food imports. Up to 2017, the value of food imports to total merchandise exports increased, from 26% in 2004–2006 to 42% in 2015–2017 (Fig. 8). As such, food security became increasingly dependent on other economic sectors.

Jordan is experiencing absolute water scarcity and has thus very limited options to expand its agricultural production. Its water availability is among the lowest in the world (Jouhari, 2018) with 95 m^3^/capita/year in 2017 (FAO, 2022b) and the lower Jordan river basin is closed (Venot et al., 2008). Most groundwater aquifers are overexploited (Al-Shibli et al., 2017). To augment the water budget, Jordan is attempting to construct the Red Sea–Dead Sea Water Conveyance to provide desalinated water to the city of Amman mainly for domestic use (Alqadi & Kumar, 2014; Rajsekhar & Gorelick, 2017). National policies promote wastewater reuse in agriculture as a water savings measure, although gains in wastewater reuse are virtual as they come at the expense of reduced groundwater resources and environmental river flows. Transboundary water challenges involving Israel, Syria and Lebanon related to the Hasbani River and Lake Tiberias have already resulted in reduced flows into Jordan (Hussein, 2019; Müller et al., 2017; The World Bank, 2017). These developments underline the limited scope for sustainable water supply enhancements and agricultural expansion for food production in Jordan (more information in Table viii in S.I.).

### Comparative analysis—different countries, but a shared narrative

In this section we discuss and compare the food security strategies of Egypt and Jordan as evolved before, during and after the 2008 food crisis.

Egypt attempted to reduce the impact of the 2008 food crisis by controlling prices, subsidizing both production and consumption. Production subsidies were a key component of this strategy, as over half of demand was met through domestic production. Any reduction in production had to be compensated by increased imports, at the expense of the trade balance (Fig. 3). Consumption subsidies were applied to secure affordable access of imported food to the growing population. This strategy was temporarily successful. It initially absorbed the price increases and led to increased cereal consumption per capita, safeguarding food accessibility (Fig. ii in SI). However, Egypt was unable to continue these subsidies and domestic food production and consumption subsequently dropped after 2012 and fluctuated ever since (Fig. i in SI). Though at the end of the study period domestic production still provided for slightly more than half of national demand, the self-sufficiency ratio of cereals (domestic production to domestic consumption) dwindled fast between 2000 and 2018 (Fig. i in SI). Any further reduction in domestic cereal production will increase Egypt’s vulnerability to price volatility on the global market.

Jordan, while contextually and conditionally worse off than Egypt, due to more severe water scarcity and greater, almost full, dependence on cereal imports (Fig. 5), deployed a different strategy. The price hikes of 2008 were directly transferred to a rising cost of food, resulting in a stark food price index jump of 20% for 2006–2008 (Fig. 6), affecting food accessibility. A steep decline in national consumption of cereals was subsequently observed between 2007 and 2010, indicating a shock in food security from which the country has not been able to structurally recover (Fig. vi in SI). Although Jordan remained vulnerable to price volatility, the food price index has followed global trends since the adjustment in 2008 (Fig. 6). However, as an increasing share of economic benefits is dedicated to food imports, the country’s vulnerability to future price shocks on the global cereals market has markedly increased (Fig. 7).

The food security strategies in Egypt and Jordan contain many similarities. Both countries depend for a large (Egypt) and very large (Jordan) share of their food security on food imports, with Egypt producing about 50% of its domestic cereals and Jordan 5%. Both countries are thus highly dependent on the global market to purchase their food imports. The agricultural sector in both countries is, in economic terms, not large enough to fund the required food imports. Both countries’ food security thus fully depends on the wider economy’s capacity to finance the needed food imports at affordable prices. The food imports have taken a large share of the total merchandise exports, up to 40–45% for both countries. The attainment of food security thus came at the expense of a worsening trade balance and rising prices. This means that both economies have reduced their capacities to absorb future increases in food imports (either through risen demands or price hikes), and that their food security strategy has become more vulnerable. The outlook is bleak for both countries as they are strongly dependent on an increasingly volatile global market with volatile prices and trading volumes, whereas their own buffers to finance food imports have decreased and food demands are likely to increase further with their rising populations. Both countries are thus increasingly vulnerable to global price shocks and market volatilities that directly affect food availability and affordability.

Despite these similarities between Egypt and Jordan, there are some profound differences in the economic capacity to cope with possible price volatilities. First, the Jordan economy has better absorbed the costs of rising food imports than the Egyptian economy, as the food consumer price index in Egypt increased for 2000–2018 starker than Jordan and global trends in the same period. Second, understanding the import dependence and the value of food imports over the total merchandise export value in combination, it is clear that Egypt is trapped by its domestic cereal production which limits options in shifting to higher value crops for exports instead of cereals. With over 50% of national demand for cereals met by domestic supply (Fig. i in S.I.) and a merchandise export value dependency ratio of 45% (Fig. 4) to finance its cereal imports, Egypt can ill afford to lose any domestic cereal production capacity in the current Egyptian economy as (ceteris paribus) a 95% import dependency would require a 90% allocation of merchandise value. The steep hike in food prices from 2016 onwards (Fig. 3) has incited producers to shift towards higher value crops and sectors. This is already discernible in diminished cereal production (Fig. 1). Jordan, in contrast, increased its exports of fruits and vegetables up to 2013. However, this strategy has proven vulnerable to political-economic volatility affecting global markets as export routes were closed due to the war in Syria. For Egypt, the strategy outlined above does not apply.

## Discussion

Our findings are in line with previous research on Egypt’s worsening food security situation (Abdelaal & Thilmany, 2019; Abdelkader et al., 2018; Hashem, 2020). Although some studies regard efficiency and productivity gains as a viable solution for food security (Abdelaal & Thilmany, 2019; Alobid & Derardja, 2021), options in this direction seem very limited considering the closed status of the Nile basin and the already high efficiency at system level of 80 per cent. The paper by Abdelkader and Elshorbagy (2021) showed maximizing food self-sufficiency comes at the expense of increased agricultural water use whereas water resources are already very limited, which is in line with our argument. The paper recognizes opportunities in changes in cropping patterns (shifting to water-saving and higher value crops) that could potentially optimize the agricultural sector under water scarcity. However, dependency on higher value exports is vulnerable to other socio-political shocks which are hard to predict, as the case of Jordan shows. Moreover, as our analysis clearly indicates the agricultural trade deficit in both Jordan & Egypt is huge (on average at around 9 and 2.2 billion USD for Egypt and Jordan respectively between 2010–2018). Though higher value exports may alleviate this deficit to a small degree, it will never be able to fully bridge the deficit. Indicating that food security in these two countries largely depend on imports provided for by the wider economy. In the case of Egypt, this is further restricted, as any decrease in national cereal production will increase its dependency on imports.

Our study supports the insight that Jordan is increasingly dependent on food imports and vulnerable to price hikes, despite efforts to increase domestic food production since 2008 (Babar & Kamrava, 2014). Nevertheless, Jordan was able to keep food prices in line with the global trend of food price increases. In contrast, Egypt tried to keep price hikes below the global trend at great economic cost – this has now translated into a very steep price hike far exceeding the global average. In terms of water resources for food production, Alqadi and Kumar (2014) presented desalination as the’ optimal, if not the only, resolution of the problem’ (p. 332) for Jordan. However, the use of desalinated water for agricultural is currently limited due to the high economic cost (Burn et al., 2015). There are some cases where desalinated water has been used for high value crops (FAO, 2012).

Land grabbing as a food security strategy is not taken into account due to its dubious nature. This strategy involves acquiring land outside the country borders for own production. Oil rich countries, as well as countries such as South Korea and China have adapted such approach and the effects of the increase in investments have been well studied in the land-grab studies (Daniel, 2011; Kajenthira Grindle et al., 2015; Zoomers, 2010). These primarily serve to secure the national food security strategy of the investing countries by increasing cereal production abroad, and disengage these investor countries from their dependency of the global food and cereal markets and trade (e.g. global trade is substituted by direct foreign investment). Production of these new areas will only reach the global and regional markets in times of excess, when the national interests of investor countries have been satisfied. Though highly effective from an investor’s perspective, these are non-options for countries like Egypt (even though there is some evidence that Egypt is engaged in land grabbing in Mauritania and Sudan^2^ or Jordan to engage with as they lack the financial capacity to do so.

Our analysis is limited in several ways. First, the daily impact of food insecurity on people is not explored. Through the CPI and the domestic consumption per 1,000 persons some insights are gained on food accessibility. However, additional indicators on food accessibility could reveal the food insecurity of poor households. A common indicator for food accessibility is the share of food expenditures (FAO et al., 2013). Higher percentages of income spent on food are often linked to more severe experienced food insecurity, due to reduced access to food (FAO et al., 2013). In 2008, Egyptians and Jordanians spent on average 38.3% and 40.8%, respectively, of their income on food, while in oil-rich UAE, Qatar and Kuwait people spent only 9.0%, 12.8% and 14.6%, respectively (Harrigan, 2012). Second, our analysis does not consider meat and dairy and thus it is limited in providing a complete assessment food security and nutrition. Lastly, the Covid-19 crisis has re-emphasised the vulnerability to shocks associated with reliance on global markets. The pandemic has hampered global and domestic trade, with some countries moving to protect their own markets and production, raising the spectre of increased prices and hunger (Falkendal et al., 2021; Sulser & Dunston, 2020), and putting an unexpected additional pressure on global food security strategies. Fuller impacts of the pandemic on international trade are expected to emerge as the health emergency recedes and lessons learned come to light (Kerr, 2020).

## Conclusions and recommendations for policy

The aim of this paper was to compare the food security strategies of Egypt and Jordan, before, during and after the 2008 world food price crisis. Our analysis reveals the limited options available to water-scarce countries to respond to growing food demand in case they lack a strong exporting sector, such as oil countries, to finance food imports. Water scarcity is expected to increase even further due to population growth, economic development and climate change. Food production in water scarce areas will thus be further constrained. In these water scarce areas food security can only be achieved through food imports.

Whereas seemingly both countries are in a same predicament (delimited water resources and outpacing food demands), their outlook for the future is starkly different. Any future increases in food demand will have to be met by food imports that are increasingly susceptible to price volatilities and hikes (due to climate change and political instability). The countries’ economic capacity to cope with such volatilities and hikes, is very different as our analysis shows. Jordan has a cereal food import dependence of 95% that is financed by 45% of its merchandise export value. Egypt, in contrast, has a food import dependence of 50% also financed by 45% of its merchandise export. This showcases the difficulty for Egypt to shift its food security to a higher import dependence as a 95% import dependence would require (ceteris paribus) a 90% allocation of merchandise value. Egypt is thus trapped within its domestic food supply capacity and has little economic room to manoeuvre to further shift its food security to a higher import dependence. In other words, Egypt depends on low value domestic cereal production to keep import dependence affordable. The dire situation of Egypt is further imperilled by: i) the increasing risks of climate change and water availability induced failures in domestic cereal production, ii) further reduction of water availability due to upstream interventions in the Nile, and iii) the extreme price hikes it has endured in the Consumer Price Index. Any further shock in the food system will be translated into higher food prices and lower availability. Thus, the current food system in Egypt seems to be stretched to the limit. Jordan is susceptible as well, but primarily to global price fluctuations and food availability, and to the economic performance of its wider economy to absorb these fluctuations. With the Consumer Price Index at global average, it still has some capacity to absorb short-term hikes.

Development of other economic sectors in these water scarce countries, particularly the services and industries sectors, will increase their demand on the very limited water resource base and likely involve redistributions of agricultural water to other economic sectors. This will likely reduce the domestic agricultural production and therefore countries will need to increasingly base their food security on the global trade strategy. Increases in import prices and market volatilities might make this option less attractive in the future, especially if more high-value crops are produced, and global cereal production declines. This poses a two-fold risk that decision-makers face. First, export prices for high-value crops might decline, as production of these commodities increases. Second, cereal import prices might rise, if a drop in global production materializes. Diversification of the export portfolio, ensuring a diverse range of agricultural products to export, could alleviate part of this risk.

Following the conceptualization of Clapp (2015, 2017) which advocates for viewing the food self-sufficiency strategy and the reliance on global markets through imports as a continuum, our analysis shows that Egypt is positioned in the middle of such continuum while Jordan is positioned towards reliance on global markets. In times of crisis (climate change resulting in climatic shocks, world food crises such as the one experienced in 2008, COVID-19 or the recent Ukrainian war), responding to the food security question cannot be bound to one specific strategy but rather on a variety of approaches that co-exist in order to spread the risks associated with each approach. These approaches range from national and/or interregional grain reserves (IATP, 2012) to influencing food consumption patterns and reduction of food losses (Terwisscha van Scheltinga et al., 2021).

In the past, national grain reserve programmes were implemented to safeguard global price stability. Though these were abolished in the era of structural adjustment, they have been revisited since the price hikes of 2008 (IATP, 2012). In addition, interregional grain reserves have also been adapted for specific cases in Southeast Asia (ASEAN), West Africa (ECOWAS) and Southern Africa (SADC) (IATP, 2012). In light of climate change impacts on global food production and the projected increases in production variability it may be time to rethink and re-evaluate such a strategy. The capacity of both Egypt and Jordan to absorb and cope with future global price hikes and shocks is rapidly decreasing as our analysis has shown. The repercussions of these will be felt, first and foremost, by the rural and urban poor, but may well have wider socio-economic impacts in the region and beyond, as we saw after the crises of 2008 and 2011. National and interregional grain reserves may then offer access to grain at an affordable price.

## Supplementary Information

Below is the link to the electronic supplementary material.

Supplementary file1 (DOCX 100 KB)

## Footnotes

1 The Water Productivity Improvement in Practice (WaterPIP) project facilitates increased crop water productivity in countries in Africa and the Near East and Northern Africa (NENA) region. For more information about Water-PIP, visit https://waterpip.un-ihe.org/welcome-waterpip.

2 More information on the land grabbing endeavours of countries can be found in the Land Matrix database, at https://landmatrix.org/map.
