# Supplementary material for: Food security under water scarcity: a comparative analysis of Egypt and Jordan
Source: Food Secur. 2022 Sep 16;15(1):171–85. doi: 10.1007/s12571-022-01310-y (PMC9483414; doi:10.1007/s12571-022-01310-y)
Supplement: Supplementary file 1 — Supplementary file1 (DOCX 100 KB) [file 12571_2022_1310_MOESM1_ESM.docx]

**Food security under water scarcity: cases of Egypt and Jordan - Supplementary Material**

Maria Christoforidou^a*^, Gerlo Borghuis^a^, Chris Seijger^a^, Gerardo E. van Halsema^a^, Petra Hellegers^a^

^a^ Water Resources Management Group, Wageningen University and Research, Wageningen, The Netherlands

*Corresponding author, maria.christoforidou@wur.nl

Table i – General Population and Agricultural Trade Information for Egypt (source: FAO, 2022a)

| Year | Population (million people) | Total Agricultural Trade Balance (million USD) | Food Consumer Price Index (CPI) (2015=100) for **Egypt** for January of each year | Food Consumer Price Index (CPI) (2015=100) for **World** for January of each year | Value of food imports in total merchandise exports (%) | |
| --- | --- | --- | --- | --- | --- | --- |
|  | **Element**: Total Population - both sexes | **Element**: Import value and Export Value **Item**: Agricultural Products + (Total) Calculated as export value - import value | **Item**: Consumer Prices, Food Indices (2015=100) | **Item**: Consumer Prices, Food Indices (2015=100) | Item: Value of food imports in total merchandise exports (percent) (3-year average) | |
| 2000 | 68.83 | -3014.18 | 27.72 | 59.47 | *3-year period* | *Value (%)* |
| 2001 | 70.15 | -2717.05 | 28.01 | 61.19 | 2000-2002 | 40 |
| 2002 | 71.49 | -2699.99 | 28.38 | 63.33 | 2001-2003 | 34 |
| 2003 | 72.83 | -1843.40 | 29.64 | 64.41 | 2002-2004 | 29 |
| 2004 | 74.17 | -1809.42 | 31.15 | 66.84 | 2003-2005 | 25 |
| 2005 | 75.52 | -2841.79 | 28.11 | 68.43 | 2004-2006 | 23 |
| 2006 | 76.87 | -3453.25 | 29.65 | 70.23 | 2005-2007 | 25 |
| 2007 | 78.23 | -4728.44 | 32.72 | 72.43 | 2006-2008 | 27 |
| 2008 | 79.64 | -6775.90 | 37.29 | 77.48 | 2007-2009 | 30 |
| 2009 | 81.13 | -4346.64 | 44.87 | 82.33 | 2008-2010 | 33 |
| 2010 | 82.76 | -8815.28 | 51.58 | 82.80 | 2009-2011 | 37 |
| 2011 | 84.53 | -9626.98 | 61.34 | 86.47 | 2010-2012 | 41 |
| 2012 | 86.42 | -11111.86 | 68.18 | 90.89 | 2011-2013 | 42 |
| 2013 | 88.40 | -8940.97 | 73.54 | 93.74 | 2012-2014 | 43 |
| 2014 | 90.42 | -9830.13 | 87.24 | 96.30 | 2013-2015 | 44 |
| 2015 | 92.44 | -8739.83 | 92.27 | 99.27 | 2014-2016 | 43 |
| 2016 | 94.45 | -7441.00 | 104.61 | 101.48 | 2015-2017 | 43 |
| 2017 | 96.44 | -8291.96 | 143.57 | 103.28 |  |  |
| 2018 | 98.42 | -9217.36 | 167.76 | 106.00 |  |  |

Table ii – Cereal analysis for Egypt (source: FAO, 2022a)

| Year | Cereals Production (million tonnes) | Imports (million tonnes) | Exports  (million tonnes) | Import Value (million USD) | Export Value (million USD) | Trade Balance  (million USD) | Domestic Consumption (million tonnes) | Self-sufficiency Ratio (SSR) (%) | Domestic Consumption per 1000 people (tonnes/1000 people) |
| --- | --- | --- | --- | --- | --- | --- | --- | --- | --- |
|  | **Element**: production quantity **Item**: Cereals, Total + (Total) | **Element**: Import quantity **Item**: Cereals + (Total) | **Element**: Export quantity **Item**: Cereals + (Total) | **Element**: Import value **Item**: Cereals + (Total) | **Element**: Export value **Item**: Cereals + (Total) | Calculated as  export value - import value | Calculated as production + imports - exports | Calculated as production/domestic consumption | Calculated as domestic consumption/population |
| 2000 | 20.11 | 9.65 | 0.40 | 1264.73 | 113.64 | -1151.09 | 29.37 | 68% | 426.62 |
| 2001 | 18.56 | 9.35 | 0.68 | 1274.30 | 139.31 | -1134.99 | 27.23 | 68% | 388.13 |
| 2002 | 20.19 | 10.32 | 0.48 | 1412.76 | 107.90 | -1304.87 | 30.04 | 67% | 420.22 |
| 2003 | 20.68 | 8.12 | 0.62 | 1137.62 | 155.59 | -982.03 | 28.18 | 73% | 386.97 |
| 2004 | 20.82 | 6.82 | 0.84 | 1096.70 | 233.36 | -863.34 | 26.80 | 78% | 361.27 |
| 2005 | 22.42 | 10.89 | 1.15 | 1636.60 | 319.56 | -1317.05 | 32.16 | 70% | 425.86 |
| 2006 | 22.52 | 11.90 | 1.03 | 1952.10 | 311.55 | -1640.55 | 33.39 | 67% | 434.36 |
| 2007 | 21.60 | 13.63 | 1.25 | 3272.22 | 410.12 | -2862.10 | 33.98 | 64% | 434.33 |
| 2008 | 23.74 | 12.36 | 0.32 | 3509.14 | 197.42 | -3311.72 | 35.78 | 66% | 449.26 |
| 2009 | 22.76 | 14.58 | 0.77 | 3173.36 | 524.53 | -2648.82 | 36.57 | 62% | 450.71 |
| 2010 | 19.46 | 16.89 | 0.70 | 3899.60 | 410.20 | -3489.39 | 35.65 | 55% | 430.77 |
| 2011 | 22.01 | 17.00 | 0.22 | 5455.06 | 78.63 | -5376.43 | 38.80 | 57% | 458.97 |
| 2012 | 23.76 | 17.85 | 0.27 | 5808.59 | 145.96 | -5662.64 | 41.33 | 57% | 478.24 |
| 2013 | 24.12 | 16.15 | 0.44 | 4744.18 | 242.58 | -4501.60 | 39.83 | 61% | 450.58 |
| 2014 | 23.32 | 19.47 | 0.25 | 5027.57 | 79.74 | -4947.83 | 42.55 | 55% | 470.53 |
| 2015 | 23.14 | 18.72 | 0.46 | 3951.50 | 178.63 | -3772.88 | 41.41 | 56% | 447.91 |
| 2016 | 23.39 | 19.78 | 0.40 | 3729.41 | 120.14 | -3609.27 | 42.77 | 55% | 452.80 |
| 2017 | 22.92 | 18.56 | 0.39 | 4416.37 | 108.09 | -4308.28 | 41.10 | 56% | 426.15 |
| 2018 | 22.05 | 16.88 | 0.39 | 4561.92 | 176.56 | -4385.36 | 38.54 | 57% | 391.57 |

Table iii – Fruit and Vegetables analysis for Egypt (source: FAO, 2022a)

| Year | Fruit Production (million tonnes) | Vegetables Production (million tonnes) | Fruits and Vegetables Production (million tonnes) | Imports (million tonnes) | Exports  (million tonnes) | Import Value (million USD) | Export Value (million USD) | Trade Balance  (million USD) | Domestic Consumption (million tonnes) | Self-sufficiency Ration (SSR) (%) | Domestic Consumption per 1000 people (tonnes/1000 people) |
| --- | --- | --- | --- | --- | --- | --- | --- | --- | --- | --- | --- |
|  | **Element**: production quantity **Item**: Fruits Primary + (Total) | **Element**: production quantity **Item**: Vegetables Primary + (Total) | Calculated as fruits + vegetables | **Element**: Import quantity **Item**: Fruit and Vegetables + (Total) | **Element**: Export quantity **Item**: Fruit and Vegetables + (Total) | **Element**: Import value **Item**: Fruit and Vegetables + (Total) | **Element**: Export value **Item**: Fruit and Vegetables + (Total) | Calculated as  export value - import value | Calculated as production + imports - exports | Calculated as production/domestic consumption | Calculated as domestic consumption/population |
| 2000 | 9.55 | 12.57 | 22.12 | 0.66 | 0.53 | 262.05 | 133.30 | -128.75 | 22.25 | 0.99 | 323.29 |
| 2001 | 9.66 | 11.77 | 21.43 | 0.88 | 0.77 | 318.84 | 165.83 | -153.01 | 21.53 | 1.00 | 306.89 |
| 2002 | 9.91 | 12.46 | 22.37 | 0.88 | 0.81 | 329.83 | 169.13 | -160.71 | 22.44 | 1.00 | 313.91 |
| 2003 | 9.93 | 13.61 | 23.54 | 0.87 | 0.95 | 301.36 | 205.97 | -95.39 | 23.45 | 1.00 | 322.05 |
| 2004 | 10.34 | 14.02 | 24.36 | 0.75 | 1.26 | 292.11 | 335.88 | 43.77 | 23.86 | 1.02 | 321.64 |
| 2005 | 11.02 | 14.41 | 25.43 | 1.28 | 1.23 | 481.26 | 365.12 | -116.14 | 25.48 | 1.00 | 337.37 |
| 2006 | 12.05 | 15.55 | 27.60 | 1.32 | 1.25 | 435.25 | 367.93 | -67.32 | 27.67 | 1.00 | 359.91 |
| 2007 | 12.14 | 16.25 | 28.39 | 1.73 | 1.35 | 751.76 | 590.77 | -160.99 | 28.77 | 0.99 | 367.78 |
| 2008 | 12.03 | 17.30 | 29.33 | 1.85 | 1.42 | 982.10 | 1004.91 | 22.82 | 29.75 | 0.99 | 373.62 |
| 2009 | 12.39 | 18.78 | 31.17 | 1.93 | 2.14 | 1271.17 | 1894.24 | 623.08 | 30.96 | 1.01 | 381.55 |
| 2010 | 12.31 | 16.77 | 29.09 | 2.73 | 1.91 | 1478.28 | 1371.46 | -106.82 | 29.91 | 0.97 | 361.39 |
| 2011 | 12.47 | 16.44 | 28.91 | 2.68 | 3.11 | 1901.64 | 2159.65 | 258.02 | 28.48 | 1.02 | 336.88 |
| 2012 | 13.50 | 16.94 | 30.44 | 2.74 | 2.02 | 2110.09 | 1910.28 | -199.81 | 31.15 | 0.98 | 360.46 |
| 2013 | 13.26 | 15.10 | 28.37 | 2.58 | 2.92 | 2039.57 | 2359.24 | 319.67 | 28.03 | 1.01 | 317.01 |
| 2014 | 14.79 | 16.34 | 31.13 | 3.05 | 3.45 | 2140.42 | 2305.28 | 164.85 | 30.73 | 1.01 | 339.83 |
| 2015 | 15.42 | 16.39 | 31.81 | 3.00 | 3.48 | 1866.18 | 2346.29 | 480.11 | 31.32 | 1.02 | 338.82 |
| 2016 | 15.16 | 15.29 | 30.45 | 2.96 | 3.47 | 1716.16 | 2317.18 | 601.02 | 29.95 | 1.02 | 317.07 |
| 2017 | 14.90 | 15.51 | 30.41 | 1.79 | 3.59 | 1730.89 | 2721.75 | 990.86 | 28.61 | 1.06 | 296.61 |
| 2018 | 15.15 | 15.57 | 30.73 | 2.74 | 3.40 | 2387.70 | 2748.77 | 361.07 | 30.07 | 1.02 | 305.49 |

Table iv – General Population and Agricultural Trade Information for Jordan (source: FAO, 2022a)

| Year | Population (million people) | Total Agricultural Trade Balance (million USD) | Food Consumer Price Index (CPI) (2015=100) for **Jordan** for January of each year | Food Consumer Price Index (CPI) (2015=100) for **World** for January of each year | Value of food imports in total merchandise exports (%) | |
| --- | --- | --- | --- | --- | --- | --- |
|  | **Element**: Total Population - both sexes | **Element**: Import value and Export Value Item: Agricultural Products + (Total) Calculated as export value - import value | **Item**: Consumer Prices, Food Indices (2015=100) | **Item**: Consumer Prices, Food Indices (2015=100) | **Item**: Value of food imports in total merchandise exports (percent) (3-year average) | |
| 2000 | 5.12 | -545.97 | 52.78 | 59.47 | *3-year period* | *Value (%)* |
| 2001 | 5.22 | -525.71 | 52.93 | 61.19 | 2000-2002 | 30 |
| 2002 | 5.32 | -454.83 | 53.08 | 63.33 | 2001-2003 | 27 |
| 2003 | 5.43 | -576.30 | 53.29 | 64.41 | 2002-2004 | 28 |
| 2004 | 5.58 | -814.24 | 54.77 | 66.84 | 2003-2005 | 28 |
| 2005 | 5.77 | -750.11 | 57.30 | 68.43 | 2004-2006 | 26 |
| 2006 | 5.99 | -811.94 | 60.18 | 70.23 | 2005-2007 | 27 |
| 2007 | 6.26 | -1249.74 | 67.73 | 72.43 | 2006-2008 | 28 |
| 2008 | 6.56 | -1723.79 | 73.82 | 77.48 | 2007-2009 | 30 |
| 2009 | 6.89 | -1308.19 | 83.74 | 82.33 | 2008-2010 | 30 |
| 2010 | 7.26 | -1431.03 | 85.37 | 82.80 | 2009-2011 | 31 |
| 2011 | 7.66 | -1994.84 | 89.38 | 86.47 | 2010-2012 | 34 |
| 2012 | 8.09 | -2172.94 | 92.50 | 90.89 | 2011-2013 | 37 |
| 2013 | 8.52 | -2097.48 | 97.04 | 93.74 | 2012-2014 | 39 |
| 2014 | 8.92 | -2301.13 | 99.46 | 96.30 | 2013-2015 | 40 |
| 2015 | 9.27 | -2237.37 | 100.92 | 99.27 | 2014-2016 | 42 |
| 2016 | 9.55 | -2713.11 | 98.47 | 101.48 | 2015-2017 | 42 |
| 2017 | 9.79 | -2497.71 | 97.08 | 103.28 |  |  |
| 2018 | 9.97 | -2537.45 | 95.72 | 106.00 |  |  |

Table v – Cereal analysis for Jordan (source: FAO, 2022a)

| Year | Cereals Production (million tonnes) | Imports (million tonnes) | Exports  (million tonnes) | Import Value (million USD) | Export Value (million USD) | Trade Balance  (million USD) | Domestic Consumption (million tonnes) | Self-sufficiency Ration (SSR) (%) | Domestic Consumption per 1000 people (tonnes/1000 people) |
| --- | --- | --- | --- | --- | --- | --- | --- | --- | --- |
|  | **Element**: production quantity **Item**: Cereals, Total + (Total) | **Element**: Import quantity **Item**: Cereals + (Total) | **Element**: Export quantity **Item**: Cereals + (Total) | **Element**: Import value **Item**: Cereals + (Total) | **Element**: Export value **Item**: Cereals + (Total) | Calculated as  export value - import value | Calculated as production + imports - exports | Calculated as production/domestic consumption | Calculated as domestic consumption/population |
| 2000 | 0.06 | 1.54 | 0.00 | 239.80 | 0.00 | -239.80 | 1.59 | 4% | 310.38 |
| 2001 | 0.05 | 1.53 | 0.01 | 232.94 | 0.00 | -232.94 | 1.57 | 3% | 301.57 |
| 2002 | 0.11 | 1.53 | 0.00 | 217.58 | 0.00 | -217.58 | 1.64 | 7% | 308.20 |
| 2003 | 0.08 | 1.97 | 0.04 | 251.72 | 0.01 | -251.71 | 2.01 | 4% | 369.88 |
| 2004 | 0.05 | 2.14 | 0.02 | 391.29 | 0.00 | -391.29 | 2.18 | 2% | 390.60 |
| 2005 | 0.10 | 1.94 | 0.00 | 352.30 | 0.00 | -352.30 | 2.04 | 5% | 353.43 |
| 2006 | 0.06 | 1.94 | 0.01 | 341.61 | 0.00 | -341.60 | 1.99 | 3% | 331.90 |
| 2007 | 0.05 | 2.56 | 0.01 | 679.46 | 0.00 | -679.45 | 2.60 | 2% | 415.66 |
| 2008 | 0.05 | 2.23 | 0.02 | 897.80 | 0.01 | -897.79 | 2.26 | 2% | 344.28 |
| 2009 | 0.06 | 1.86 | 0.05 | 544.53 | 0.01 | -544.52 | 1.87 | 3% | 271.01 |
| 2010 | 0.09 | 1.70 | 0.01 | 511.70 | 0.00 | -511.69 | 1.77 | 5% | 243.83 |
| 2011 | 0.08 | 2.26 | 0.02 | 847.73 | 0.01 | -847.72 | 2.32 | 4% | 302.73 |
| 2012 | 0.08 | 2.46 | 0.03 | 903.58 | 0.01 | -903.56 | 2.52 | 3% | 311.33 |
| 2013 | 0.10 | 2.13 | 0.03 | 786.11 | 0.01 | -786.10 | 2.20 | 5% | 258.80 |
| 2014 | 0.09 | 2.77 | 0.04 | 917.72 | 0.01 | -917.71 | 2.82 | 3% | 316.61 |
| 2015 | 0.10 | 2.34 | 0.10 | 717.71 | 0.03 | -717.68 | 2.33 | 4% | 251.87 |
| 2016 | 0.10 | 3.88 | 0.01 | 942.75 | 0.00 | -942.75 | 3.97 | 3% | 415.07 |
| 2017 | 0.10 | 2.82 | 0.08 | 693.76 | 0.02 | -693.74 | 2.84 | 3% | 289.92 |
| 2018 | 0.08 | 3.00 | 0.48 | 824.41 | 0.11 | -824.30 | 2.60 | 3% | 260.63 |

Table vi – Fruit and Vegetables analysis for Jordan (source: FAO, 2022a)

| Year | Fruit Production (million tonnes) | Vegetables Production (million tonnes) | Fruits and Vegetables Production (million tonnes) | Imports (million tonnes) | Exports  (million tonnes) | Import Value (million USD) | Export Value (million USD) | Trade Balance  (million USD) | Domestic Consumption (million tonnes) | Self-sufficiency Ration (SSR) (%) | Domestic Consumption per 1000 people (tonnes/1000 people) |
| --- | --- | --- | --- | --- | --- | --- | --- | --- | --- | --- | --- |
|  | **Element**: production quantity **Item**: Fruits Primary + (Total) | **Element**: production quantity **Item**: Vegetables Primary + (Total) | Calculated as fruits + vegetables | **Element**: Import quantity **Item**: Fruits + (Total) and Vegetables + (Total) | **Element**: Export quantity **Item**: Fruits + (Total) and Vegetables + (Total) | **Element**: Import value **Item**: Fruit + (Total) and Vegetables + (Total) | **Element**: Export value **Item**: Fruit + (Total) and Vegetables + (Total) | Calculated as  export value - import value | Calculated as production + imports - exports | Calculated as production/domestic consumption | Calculated as domestic consumption/population |
| 2000 | 0.31 | 0.80 | 1.11 | 0.09 | 0.40 | 52.26 | 101.19 | 48.93 | 0.80 | 139% | 155.45 |
| 2001 | 0.37 | 0.66 | 1.03 | 0.10 | 0.41 | 56.97 | 131.08 | 74.11 | 0.72 | 143% | 138.43 |
| 2002 | 0.41 | 0.95 | 1.37 | 0.10 | 0.43 | 59.87 | 146.55 | 86.68 | 1.04 | 131% | 195.65 |
| 2003 | 0.41 | 0.89 | 1.29 | 0.11 | 0.44 | 62.31 | 166.34 | 104.03 | 0.96 | 134% | 177.47 |
| 2004 | 0.40 | 1.07 | 1.46 | 0.13 | 0.51 | 71.40 | 191.91 | 120.51 | 1.09 | 135% | 194.64 |
| 2005 | 0.41 | 1.25 | 1.66 | 0.15 | 0.58 | 88.88 | 265.02 | 176.13 | 1.23 | 135% | 213.83 |
| 2006 | 0.43 | 1.18 | 1.61 | 0.16 | 0.60 | 103.62 | 267.79 | 164.18 | 1.17 | 138% | 195.70 |
| 2007 | 0.35 | 1.22 | 1.57 | 0.19 | 0.78 | 132.92 | 436.63 | 303.71 | 0.98 | 161% | 156.25 |
| 2008 | 0.38 | 1.19 | 1.57 | 0.21 | 0.78 | 162.25 | 474.11 | 311.86 | 1.00 | 157% | 152.19 |
| 2009 | 0.40 | 1.29 | 1.69 | 0.23 | 0.87 | 190.84 | 487.13 | 296.29 | 1.06 | 159% | 154.19 |
| 2010 | 0.48 | 1.41 | 1.89 | 0.23 | 0.79 | 190.31 | 514.14 | 323.83 | 1.33 | 142% | 183.26 |
| 2011 | 0.47 | 1.57 | 2.04 | 0.25 | 0.89 | 227.62 | 580.16 | 352.54 | 1.40 | 146% | 183.26 |
| 2012 | 0.47 | 1.43 | 1.89 | 0.27 | 0.85 | 252.28 | 688.60 | 436.32 | 1.31 | 144% | 162.46 |
| 2013 | 0.45 | 1.60 | 2.05 | 0.29 | 0.99 | 286.37 | 704.41 | 418.04 | 1.35 | 152% | 158.85 |
| 2014 | 0.47 | 1.56 | 2.03 | 0.32 | 0.94 | 305.50 | 786.10 | 480.61 | 1.41 | 143% | 158.61 |
| 2015 | 0.59 | 1.69 | 2.28 | 0.27 | 0.84 | 318.75 | 762.73 | 443.99 | 1.71 | 134% | 184.31 |
| 2016 | 0.62 | 1.89 | 2.51 | 0.29 | 0.73 | 349.54 | 591.62 | 242.07 | 2.07 | 121% | 216.57 |
| 2017 | 0.55 | 1.43 | 1.97 | 0.27 | 0.63 | 338.57 | 551.63 | 213.06 | 1.61 | 122% | 164.67 |
| 2018 | 0.56 | 1.51 | 2.07 | 0.24 | 0.57 | 295.62 | 470.06 | 174.44 | 1.73 | 119% | 174.00 |

Fig. i - Domestic production and consumption of cereals in Egypt, alongside imports and exports, 2000-2018. Steadily increasing imports and domestic consumption and decreasing exports of cereals pre 2008, and cereal production increases and stable production post 2008 (FAO, 2022a).

Fig. ii - Cereal consumption per 1,000 persons in Egypt, 2000-2018. Slightly increasing cereal consumption per 1,000 persons up to 2016 (FAO, 2022a).

Fig. iii - Cereals and fruits and vegetables exports in Egypt, 2000-2018. Increasing cereal and fruits and vegetables exports pre 2008, and decreasing cereal exports and increasing fruits and vegetables post 2008 (FAO, 2022a).

|  | **Explanation** | **Source** |
| --- | --- | --- |
| **Total renewable water resources per capita (m^3^/capita/year)** | 596.2 (2017)  Reaching the threshold of absolute water scarcity of 500 m3/capita/year. | FAO, 2022b |
| **Possibilities for water supply enhancements** | The Nile River Basin is effectively closed as the outflow to the Mediterranean Sea consists of 5-15% of reported annual discharge into Egypt.  Groundwater (renewable and non-renewable) is already being used and plans include further increases in fossil groundwater use. However, these developments threaten the long-term sustainability of agricultural production.  Increases in Nile water reaching Egypt are unlikely due to irrigation developments in upstream countries. As Egypt’s dependency ratio is 98.26%, the country is vulnerable to upstream water management.  Egypt shows early signs of uptake of desalination in the face of reduced Nile flows. | FAO and IHE Delft, 2020  MALR, 2009; Hamza and Mason, 2004    Digna et al., 2018; FAO, 2022b  Molle, 2019 |
| **Potential for water savings** | Egypt has a long history of reuse of drainage water.  Irrigation efficiency improvements for potential water savings are unrealistic due to area expansion and the increased water shortages. | Abd Ellah, 2020  MWRI, 2017, author’s interpretation |

Table vii - Egypt’s water resources base, indicating limited options for achieving food security

Fig. iv - Cereal production in Jordan, 2000-2018. Limited and highly variable production, with steady increases post 2008 (FAO, 2022a).

Fig. v - Cereal production, imports, exports and domestic consumption in Jordan, 2000-2018. Low domestic cereal production, declining imports and consumption between 2007-2011 and increasing imports and domestic consumption (FAO, 2022a).

Fig. vi - Domestic consumption of cereals and fruits and vegetables per 1,000 persons in Jordan, 2000-2018. Decreasing staple consumption and relatively stable consumption of fruits and vegetables per 1,000 persons (FAO, 2022a).

Fig. vii - Production, imports, exports and domestic consumption of fruits and vegetables in Jordan, 2000-2018. Steady increases in production up to 2016 and decreasing exports after 2013 (FAO, 2022a).

Table viii - Jordan’s Water Resource base, indicating the limited options for achieving food security

|  | **Explanation** | **Source** |
| --- | --- | --- |
| **Total renewable water resources per capita (m3/capita/year)** | 95.75 (2017)  Well below the threshold of absolute water scarcity of 500 m3/capita/year. | FAO, 2022b |
| **Possibilities for water supply enhancements** | The Lower Jordan River basin is closed and thus no additional resources can be used.  Most aquifers are being over exploited.  Agricultural water use is capped at 700 MCM per year, clearly indicating the limited focus and applicability of increasing water supply for the agricultural sector in Jordan.  Jordan’s dependency ratio is 27.21%.  Desalination (mainly for domestic supply) is planned through the Red Sea–Dead Sea Water Conveyance project. | Venot et al., 2008  Al-Shibli et al., 2017  Ministry of Water and Irrigation, 2016  FAO, 2022b  Ministry of Water and Irrigation, 2016 |
| **Potential for water savings** | Treated wastewater (TWW) is envisioned to save water that would otherwise go unused. However, based on the water budget for 2014, TWW re-use in agriculture comes at the expense of non-renewable groundwater (see Figure S.M.10). As such, TWW use in agriculture threatens long-term groundwater sustainability. | Ministry of Water and Irrigation, 2016, authors’ calculations |

**References**

Abd Ellah, R.G., 2020. Water resources in Egypt and their challenges: Lake Nasser case study. The Egyptian Journal of Aquatic Research, 46(1), 1–12.

Al-Shibli, F.M., Maher, W.A., Thompson, R.M., 2017. The need for a quantitative analysis of risk and reliability for formulation of water budget in Jordan. Jordan Journal of Earth and Environmental Sciences, 8(2), 77–89.

Digna, R.F., Mohamed, Y.A., Van der Zaag, P., Uhlenbrook, S., Van der Krogt, W., Corzo, G., 2018. Impact of water resources development on water availability for hydropower production and irrigated agriculture of the Eastern Nile Basin. Journal of Water Resources Planning and Management. DOI: 10.1061/(ASCE)WR.1943-5452.0000912.

FAO, IHE Delft, 2020. Water accounting in the Nile River Basin. FAO WaPOR Water Accounting Reports. Rome: FAO. <https://doi.org/10.4060/ca9895en>.

FAO. (2022a). FAOSTAT database. License: CC BY-NC-SA 3.0 IGO. Extracted from: <https://www.fao.org/faostat/en/#data>. Date of Access: December 2020.

FAO. (2022b). AQUASTAT database. License: CC BY-NC-SA 3.0 IGO. Extracted from: <https://www.fao.org/faostat/en/#data>. Date of Access: December 2020

Hamza, W., Mason, S., 2004. Water availability and food security challenges in Egypt, in: Proceedings International Forum on Food Security Under Water Scarcity in the Middle East: Problems and Solutions, Como, Italy, pp. 24–27.

MALR. (2009). Sustainable agricultural development strategy towards 2030. Cairo: Ministry of Agriculture and Land Reclamation.

Ministry of Water and Irrigation. (2016). National Water Strategy 2016–2025.

Molle, F. (2019). Egypt, in: Molle, F., Sanchis-Ibor, C. and Avellà-Reus, L. (Eds.), Irrigation in the Mediterranean: Technologies, Institutions and Policies (pp. 243–277). Springer International Publishing. DOI: <https://doi.org/10.1007/978-3-030-03698-0_9>

MWRI. (2017). State of Water in Egypt 2014-2015. Ministry of Water Resources and Irrigation, May 2017
